# Clinically Precedented Protein Kinases: Rationale for Their Use in Neurodegenerative Disease

**DOI:** 10.3389/fnagi.2020.00242

**Published:** 2020-09-02

**Authors:** Caroline L. Benn, Lee A. Dawson

**Affiliations:** ^1^LoQus23 Therapeutics, Cambridge, United Kingdom; ^2^Cerevance Ltd., Cambridge, United Kingdom

**Keywords:** inhibitor, central nervous system, ATP, small molecule, clinical development, blood brain barrier

## Abstract

Kinases are an intensively studied drug target class in current pharmacological research as evidenced by the large number of kinase inhibitors being assessed in clinical trials. Kinase-targeted therapies have potential for treatment of a broad array of indications including central nervous system (CNS) disorders. In addition to the many variables which contribute to identification of a successful therapeutic molecule, drug discovery for CNS-related disorders also requires significant consideration of access to the target organ and specifically crossing the blood-brain barrier (BBB). To date, only a small number of kinase inhibitors have been reported that are specifically designed to be BBB permeable, which nonetheless demonstrates the potential for success. This review considers the potential for kinase inhibitors in the context of unmet medical need for neurodegenerative disease. A subset of kinases that have been the focus of clinical investigations over a 10-year period have been identified and discussed individually. For each kinase target, the data underpinning the validity of each in the context of neurodegenerative disease is critically evaluated. Selected molecules for each kinase are identified with information on modality, binding site and CNS penetrance, if known. Current clinical development in neurodegenerative disease are summarized. Collectively, the review indicates that kinase targets with sufficient rationale warrant careful design approaches with an emphasis on improving brain penetrance and selectivity.

## Introduction

The protein kinase gene family comprises over 500 members and constitutes approximately 2% of all human genes. Protein kinases catalyze the transfer of the terminal phosphate group of adenosine triphosphate (ATP) onto an amino acid residue (typically serine, threonine or tyrosine) within a polypeptide chain. Up to 20% of all human proteins may be modified by kinase activity which results in a functional change of the target protein by altering cellular activity, location or association with other proteins. Consequently, protein kinases regulate a range of cellular functions through the orchestrated propagation and amplification of cellular stimuli into distinct biological responses through coordinated signal transduction cascades (Chico et al., 2009). Aberrant regulation of protein kinase activity has been linked to a diverse set of disease states including cancer, inflammation, metabolic, autoimmune, and neurological disorders. For example, large scale sequencing efforts have highlighted protein kinases as one of the most frequently mutated proteins across cancer subtypes. This in turn has stimulated the pursuit of these enzymes as potential drug targets for therapeutic intervention.

**FIGURE 1:**
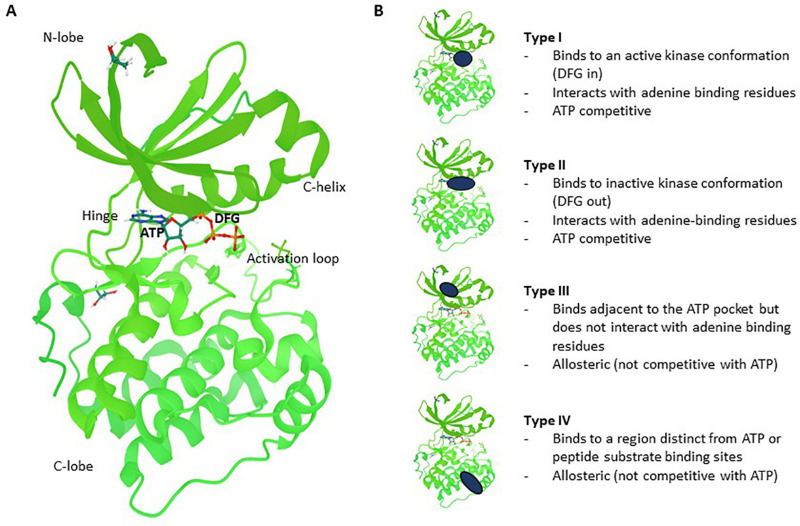
Kinase structure and different types of reversible small molecule kinase inhibitors. **(A)** Co-crystal structure of PDK1 (green) with ATP (shown in ball and sticks). Regions indicated are the N-lobe (N-terminal domain), C-lobe (C-terminal domain), C-helix, Hinge, Activation loop and the DFG regulatory motif which sits just behind part of the ATP molecule colored in orange (4RRV, PDB). **(B)** Type I inhibitors bind to the active conformation of the kinase, interact with the hinge motif and are ATP competitive. Type II binders are also ATP competitive but occupy a “back pocket” which exists in a subset of protein kinases. Type III ligands bind in a distinct hydrophobic pocket near the ATP binding site and are uncompetitive with ATP. Type IV inhibitors bind to distinct surface pockets away from the ATP binding site and are also uncompetitive with ATP. The PKD1 structure is used here to illustrate the different types of inhibitors.

The catalytic subunits of protein kinases are highly conserved and over 200 structures have been solved. Protein kinases share a common bilobal-fold comprising a predominantly beta-sheet N-terminal and a helical C-terminal domain linked by a hinge polypeptide with a canonical catalytic (and ATP binding) site residing in the cleft between these two domains (Mortenson et al., 2014). Inhibitors of protein kinases can be broadly partitioned based on their binding modes (Figure 1). Type I inhibitors bind in the ATP pocket and interact directly with the kinase “hinge motif” and are thus competitive with ATP. Type II binders are also ATP competitive and generally contain a hinge binding moiety but occupy a hydrophobic pocket beyond the “gatekeeper” residue; this pocket exists in a subset of protein kinases. Type III ligands bind in a hydrophobic pocket proximal to the ATP-binding site and inhibit via an allosteric, ATP-uncompetitive mechanism. Type IV inhibitors bind to surface pockets distinct from the ATP binding site and are thus also ATP-uncompetitive. Structural analysis of selective inhibitor complexes has elucidated deeper understanding of mechanisms and accordingly, structure-guided approaches for target-specific inhibitors have been developed for a subset of kinase targets (Murray et al., 2015; Roskoski, 2016; Heightman et al., 2018).

**FIGURE 2:**
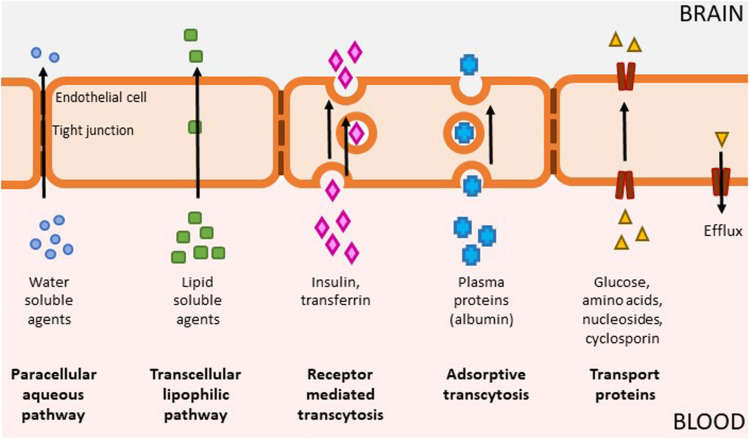
Mechanisms of BBB penetration. The brain is separated from the rest of the body by the blood-brain barrier (BBB) which is composed of capillary endothelium containing tight junctions and specific transporter systems to exclude pathogens and toxins, which unfortunately can also include therapeutic drugs. The BBB allows the passage of some molecules by several routes (1) Passive diffusion of water soluble agents through the paracellular aqueous pathway is negligible (2) Small, lipid soluble agents (e.g., nicotine, alcohol, antidepressants) can diffuse across a concentration gradient through endothelial cells via the transcellular lipophilic pathway which represents the main entry route into the brain for many currently approved therapeutics; (3) Receptor mediated transcytosis enable delivery of proteins such as insulin, transferrin, leptin, interleukins as well as nutrients such as iron and LDL; (4) Adsorptive transcytosis modulates transport of other proteins such as albumin; (5) Specialized transport proteins such as solute carriers can transport a range of substrates including glucose, amino acids, and drugs such as cyclosporine and azidothymidine. This category also includes efflux transporters, which is a key element of the BBB that can actively transport a variety of lipophilic drugs out of the endothelial cells. Brain penetration leading to exposure at the site of action, binding to the pharmacological target and expression of pharmacological activity are required for compound efficacy. Many drugs, including kinase inhibitors, have not been optimized for BBB penetration and as a result, they have reduced partitioning to the CNS. In order to achieve sufficient levels of exposure for compounds with suboptimal levels of CNS penetration, this means the level of systemic exposure will be significantly higher, which can lead to undesirable on- and off- target pharmacology.

It can thus be appreciated that kinases are eminently “druggable” using small molecule approaches but still present several challenges including ATP competition (for type 1 inhibitors), selectivity, physicochemical properties, and intellectual property. Most kinase-targeted drugs developed have been for non-CNS indications such as cancer and inflammation and have not been optimized with respect to CNS exposure. Small molecule drugs have a proven track record with respect to achieving CNS penetration, but they need to be designed with certain properties to be permeable to traverse the endothelial membranes while avoiding the efflux transporters (Figure 2). Reduced CNS penetration through the BBB can result in comparatively high systemic exposure levels in order to achieve the concurrent CNS concentrations required for efficacy – which can often lead to side effects (Figure 2). A significant proportion of the CNS-targeted kinase drugs appear to be related to neuro-oncology (Heffron, 2016). Indeed, clinical candidates with proven impact on key pathways in oncology have been leveraged with respect to systemic approaches to treating brain metastases (Franchino et al., 2018). Nevertheless, there are grounds for optimism: most kinase inhibitors have not been designed to be brain penetrant, and many screening campaigns have used compound collections loaded with “legacy” kinase inhibitor structures with suboptimal properties for CNS penetration. This situation is changing rapidly, with numerous examples now showing that it is possible to generate inhibitors that fall within a more favorable CNS property space.

## Kinase Targets Clinically Assessed for Neurodegenerative Disease Indications

This review focuses on the challenges of targeting protein kinases for CNS disease indications with in-depth analyses on selected case studies for the underlying biological rationale. Here, we discuss in depth eleven protein kinase targets that have been progressed into clinical assessment for neurodegenerative disease indications from 2008 to 2018. These comprise ABL1, CSNK1D, CSF1R, MAP3K12, GSK3B, MAPK10, LRRK2, MAPK14, MTOR, RIPK1 and ROCK, discussed in the following sections and summarised in Table 1. We consider the strength of the biological rationale (although an in-depth review of the target literature will not be undertaken here but can be found in associated reviews), small molecule inhibitors and preclinical/clinical translational efforts. Of note, very few of the molecules in the clinic at the time of writing have been rationally designed with CNS penetration in mind.

**TABLE 1:** Summary of kinases covered in manuscript with respect to function, disease association, inhibitors, and clinical development.

Kinase	Function	Disease specific rationale	Inhibitors	Clinical
ABL1	- Mediates responses to oxidative stress which has abundant linkage to disease pathophysiology.	- Substrates of kinase genetically linked to disease. - Activated ABL1 in disease tissues. - Aberrant activation leads to neurodegenerative phenotype in mice. - Genetic manipulation beneficial in models. - Pharmacological inhibition beneficial in models.	- Multiple ATP-competitive inhibitors, none designed with CNS penetration in mind. - Allosteric inhibitor not assessed.	- Furthest development: nilotinib Ph2a in PD. - No meaningful effect reported in efficacy trials.
CSNK1D	- Genetically and functionally linked to circadian rhythm (progressively altered with neurodegeneration).	- Substrates of kinase linked to disease. - Elevated level and activity reported in disease tissues. - Pharmacological inhibition beneficial in some models.	- ATP competitive inhibitors optimized for brain penetrance, have some selectivity.	- Furthest development: PF-05251749 Ph1 in healthy volunteers. - Safe and well tolerated; no efficacy assessment as yet in any disease population.
CSF1R	- Genetically and functionally linked to microglial proliferation.	- Mutations linked to AD and ALSP. - Kinase upregulated in disease tissues. - Genetic manipulation beneficial in models. - Pharmacological inhibition beneficial in models.	- Multiple ATP competitive inhibitors which have not been optimized for BBB penetration; lack of selectivity also a challenge.	- Furthest development: masitinib (non-specific inhibitor) in Ph3 for ALS and pexidartinib in Ph2a in AD.
MAP3K12/DLK	- Injury sensor that can comprise part of the JNK signaling pathway; has distinct effects depending on context.	- DLK/JNK pathway activation in disease tissue. - Genetic manipulation in models beneficial. - Pharmacological inhibition in models beneficial.	- ATP competitive inhibitors optimized for brain penetrance and have some selectivity but may be limited by toxicity.	- Furthest development: GDC-0134 in Ph1 for ALS.
GSK3β	- Multiple roles including inflammation and microtubule dynamics.	- Rare variants causative for AD and FTD. - Increased expression and activity in disease tissue. - Pharmacological inhibition beneficial in some models. - Mechanistically linked to both tau and Aβ.	- Multiple inhibitors developed including ATP competitive, non-ATP competitive, irreversible, allosteric, peptide-like – some with a degree of CNS penetration.	- Furthest development: tideglusib in Ph2a in PSP and AD, and AZD1080 in Ph1 healthy volunteers. - Toxicity may be a contributing factor limiting clinical investigations.
JNK3	- Stress signaling response (unfolded proteins, oxidative, excitotoxic, DNA damage, etc.).	- Genetic modifier for spinal and muscular atrophy. - Increased phosphorylation of kinase and substrates in disease tissues. - Genetic manipulation in models is beneficial. - Pharmacological inhibition beneficial in models.	- Multiple ATP competitive inhibitors which have good CNS penetrance, but selectivity has been more challenging. - Peptide inhibitor also promising in preclinical models.	- Furthest development: CEP1347 in Ph2a in PD, which did not show efficacy.
LRRK2	- Multiple functions including trafficking, lysosomal function.	- Genetically linked to sporadic and familial PD. - Genetic manipulation is beneficial in models. - Unclear if kinase inhibition is the correct strategy.	- Multiple ATP competitive inhibitors optimized for brain penetration. - Similar preclinical safety flags with chemically diverse molecules suggestive of on-target toxicity.	- Furthest development: DNL151 and DNL201 currently in Ph1b. - No efficacy data available yet but press releases suggest compounds are well-tolerated with no obvious toxicology.
MAPK14/p38α	- MAPK stressor signal pathway well reported in ND.	- MAPK pathway signals reported in disease tissues. - Genetic manipulation beneficial in AD models. - Pharmacological inhibition beneficial in AD models.	- Multiple ATP competitive and non-competitive molecules but not optimized for CNS penetration; selectivity is a challenge.	- Furthest development: neflamapimod in Ph2 (AD, DLB and HD), no efficacy data reported as yet.
MTOR	- Regulatory kinase for multiple functions including metabolism and mediating responses to misfolded proteins.	- Aberrant mTOR signaling in disease tissues. - Pharmacological inhibition beneficial in disease models.	- Allosteric inhibitor rapamycin (and related molecules) has good selectivity but suboptimal exposure. - Multiple ATP competitive inhibitors have suboptimal exposure and selectivity.	- Furthest development: rapamycin in Ph2 in ALS, no data has been published.
RIPK1	- Cell survival and death signaling but unclear if blocking late stage cell death will be beneficial.	- Kinase activation in disease tissue. - Pharmacological inhibition beneficial in models.	- Multiple ATP competitive and allosteric molecules; including some that have been developed to enable CNS penetration.	- Furthest development: DNL747 in Ph2 (ALS and AD), and DNL104 in Ph1 (healthy volunteers).
ROCK	- Actin cytoskeleton and axonal transport regulator; involvement in mitophagy.	- Overactivation reported in disease tissue. - Genetic manipulation beneficial in models. - Pharmacological inhibition beneficial in models.	- Multiple ATP competitive inhibitors that have poor CNS penetration and narrow safety windows.	- Furthest development: fasudil in Ph2 (ALS), results have not been reported.

### ABL1 (Abelson Murine Leukemia Viral Oncogene Homolog 1, c-Abl)

#### Biological Rationale

Oxidative stress, protein aggregate accumulation, and damaged mitochondria are common hallmarks of neurodegenerative disease and ABL1 sits at the nexus of the associated signaling pathways with roles in the regulation of receptor endocytosis, DNA repair, autophagy, cytoskeleton dynamics, cell survival, growth, and motility (Gonfloni et al., 2012). ABL1 activity is regulated by its subcellular localization, intramolecular bonds and post-translational status (in particular, its autophosphorylation at Y412 by upstream kinases) (Kharbanda et al., 1995; Shafman et al., 1997; Yuan et al., 1997; Brasher and Van Etten, 2000; Dorey et al., 2001; Ko et al., 2010; Imam et al., 2011). In adult mouse models, aberrant ABL1 activation causes neurodegeneration and neuroinflammation in forebrain neurons (Schlatterer et al., 2011). ABL1 is present in its activated form in Parkinson’s disease (PD) patients as well as in preclinical models of the disease; furthermore, ABL1 has been reported to phosphorylate α-synuclein at Y39 and the E3 ligase Parkin, both of which are genetic risk factors for PD (Ko et al., 2010; Imam et al., 2011; Schlatterer et al., 2011; Brahmachari et al., 2016; Karim et al., 2020). Deletion of the *ABL1* gene reduced α-synuclein aggregation, neuropathology, and neurobehavioral deficits and protects against MPTP (1-methyl-4-phenyl-1,2,3,6-tetrahydropyridine, a prodrug for the neurotoxin MPP+/1-methyl-4-phenylpyridinum) challenges (Ko et al., 2010; Hebron et al., 2013; Mahul-Mellier et al., 2014; Brahmachari et al., 2016). Pharmacological inhibition of ABL1 has protective effects in mouse models of PD, although these data need to be treated with caution given the likely low CNS exposures and known promiscuity of these molecules (Hebron et al., 2013; Imam et al., 2013; Karuppagounder et al., 2014; Tanabe et al., 2014; Lee et al., 2018). ABL1 hyperactivation has been shown in other neurodegenerative diseases with inhibitor treatment generally reported as beneficial for Alzheimer’s disease (AD), Niemann-Pick Type C (NPC), and amyotrophic lateral sclerosis (ALS) (Derkinderen et al., 2005; Jing et al., 2009; Klein et al., 2011; Schlatterer et al., 2011; Imamura et al., 2017; Osaki et al., 2018; Riancho et al., 2018).

#### Small Molecule Inhibitors

ABL1 oncoproteins have been a prime molecular target for cancer therapy using ATP-competitive inhibitors such as imatinib which directly challenged the viewpoint at the time that kinases were not good drug targets (Druker, 2008). Subsequent related ATP-competitive inhibitors such as nilotinib, dasatinib, bosutinib, and ponatinib were developed to address the prevalence of mutations in the ABL1 kinase domains; but all exhibit notable off-target activity including c-KIT, CSF1R, and PDGFRA/B (Shah et al., 2004; Weisberg et al., 2005; Dubreuil et al., 2009; Anastassiadis et al., 2011; Cortes et al., 2011; Ashman and Griffith, 2013; Rask-Andersen et al., 2014). Co-crystal structures of the kinase domain in complex with imatinib and other inhibitors have been critical in revealing the mechanism of action of point mutations causing drug resistance in certain cancers (Schindler et al., 2000; Nagar et al., 2002). In addition to ATP-competitive molecules, allosteric targeting of the myristate pocket and the SH2-kinase interface (a major autoinhibitory mechanism) has yielded additional compounds such as GNF-2 and asciminib (Wylie et al., 2017; Schoepfer et al., 2018). GNF-2 induces myristoylated ABL1 translocation to the endoplasmic reticulum, leading to competition with the intramolecular engagement of the NH2-terminal myristate for binding to the ABL1 kinase myristate-binding pocket.

#### Clinical Development

Initial development for ABL1 inhibitors was for indications requiring peripheral exposure and thus molecules have not been optimized for CNS penetrance. For example, it was shown preclinically that nilotinib has some penetration with 310 ng/mg of compound in brain tissue following 10 or 20 mg/kg (I.P.; 3–4 h post- dose) (Hebron et al., 2013). Recently, nilotinib (150 and 300 mg once daily for 24 weeks) was employed in an open-label clinical trial for PD and Dementia with Lewy Body (DLB; Pagan et al., 2016, 2019). The efficacy and tolerability results were promising, albeit preliminary, and constrained by the small sample size and the low doses employed (vs. oncology doses) possibly questioning the level of target engagement attained, particularly given the free brain ratio, i.e., K_*pu,u*_ of <0.1 (Lindholm et al., 2016). Recently, it was reported that while nilotinib was safe and tolerable in a PD population, it did not appear to exert a clinically meaningful effect (MJF foundation press release^1^). Bosutinib is also in the clinic for “degenerative dementias” (NCT02921477), but no brain or plasma quantification data have been reported. Arguably, bafetinib (INNO-406) may be a better molecule to test the hypothesis that c-Abl mediates PD pathogenesis given the potentially improved CNS exposure (Imam et al., 2013). K-0706 from Sun Pharma appears to represent a series of Abl inhibitors that have been assessed for CNS penetrance with a reported brain: plasma ratio, i.e., Kp of 0.4 at 1 h following a 30 mg/kg dose for a representative molecule (patent WO2017/208267). This molecule was also assessed for efficacy in a mouse model of PD and is currently being evaluated in a PD population (NCT02970019). Selectivity data on these molecules were not available.

#### Synopsis

The biological rationale for ABL1 is reasonable, particularly in PD with multiple biological substrates of the kinase being genetically linked and mechanistically connected to pathways of relevance to the disease. However, the molecules developed to date have suboptimal levels of CNS penetration and limited selectivity which may ultimately impact efficacy and tolerability since systemic exposures are likely to be in excess of those needed for CNS target engagement. Thus, more careful evaluation with suitably brain penetrant, potent, selective, and potentially better tolerated molecules may be warranted before the long-term therapeutic utility of selective ABL1 inhibitors in neurodegenerative patient populations can be fully ascertained.

### CSNK1D (Casein Kinase 1δ)

#### Biological Rationale

The casein kinase 1 family comprises seven members of constitutively active serine/threonine kinases and was first identified as a set of proteins that could readily phosphorylate casein *in vitro* (reviewed in Cozza and Pinna, 2016). Across the family there is high conservation in the kinase domain, with 98% homology between delta and epsilon but isoforms vary in the length and primary structure of the regulatory domains. Casein kinases are negatively regulated via dimer formation and phosphorylation (Knippschild et al., 2005). The casein kinase 1 family phosphorylate over 140 additional targets associated with membrane transport processes, trafficking and microtubule associated dynamics, cell cycle progression and apoptosis (Yang Y. et al., 2017; Xu et al., 2019). Mutations in *CSNK1D* have been linked to familial advanced sleep phase syndrome and acute inhibition or genetic disruption results in alterations in circadian rhythms which correlates with the observation that core clock proteins are substrates for the enzyme (Xu et al., 2005; Etchegaray et al., 2009; Isojima et al., 2009; Meng et al., 2010; Brennan et al., 2013; Kennaway et al., 2015). Furthermore, it is possible that dysregulation of circadian rhythms in AD is aligned with alterations in CSNK1D function. Elevated levels and activity of CSNK1D has been reported in AD post-mortem brain tissue and ALS (Ghoshal et al., 1999; Yasojima et al., 2000; Thal et al., 2011; Salado et al., 2014). In addition, the δ isoform has been shown to phosphorylate a range of proteins which have been genetically linked to various neurodegenerative diseases *in vitro.* However, this correlation has not consistently been corroborated *in vivo*, e.g., PS2, BACE1, Parkin, TDP43, α-synuclein, LRRK2, and tau (Walter, 2000; Walter et al., 2001; de la Torre et al., 2009; Kosten et al., 2014; Alquezar et al., 2016; Nonaka et al., 2016; Morales-Garcia et al., 2017; De Wit et al., 2018). Pharmacological modulation of CSNK1δ/ε has been reported to have beneficial impacts in ALS, FTD, and PD neurodegenerative disease phenotypes *in vivo* (Salado et al., 2014; Alquezar et al., 2016; Morales-Garcia et al., 2017).

#### Small Molecule Inhibitors

Several ATP-competitive inhibitors have been identified with a range of preferences for the δ and ε isoforms, such as the set of molecules from Pfizer which have been demonstrated to exhibit central exposure and target engagement: PF-4800567, PF-0670462, and the PET ligand PF-5236216 (Meng et al., 2010; Mente et al., 2013; Wager et al., 2014, 2017). However, true selectivity has proven to be a challenge due to the high conservation within casein kinase 1 family members and related kinases such as p38α or TTBK1/2 (Halekotte et al., 2017).

#### Clinical Development

Following preclinical development using circadian rhythm assessment as a measure of central target engagement, PF-05251749 was assessed in Phase 1 for safety and tolerability with single and multiple ascending dose studies (NCT02443740; NCT02691702). Pfizer has reported no further development since October 2018 but Biogen has recently acquired the molecule with a $75 million upfront payment presumably to pursue this further in AD populations (January 2020)^2^.

#### Synopsis

The biological rationale for this kinase in ND disorders would be significantly augmented via additional investigation in relevant *in vivo* models; particularly in TDP43-related disorders which has seen the most effort to date. Pfizer has developed molecules rationally for selectivity, potency and brain penetrance which appear to be well tolerated in the clinic as far as we can currently ascertain. Thus, it would seem further assessment of these molecules in ND populations may be warranted.

### CSF1R (Colony Stimulating Factor 1 Receptor)

#### Biological Rationale

Microglial cells have been described as the master regulators of the neuroinflammatory response associated with neurodegenerative disease. In numerous preclinical models of neurodegenerative disease, and in post-mortem brain samples from AD patients, an increase in microglia number and their proliferation rate has been observed (Perry et al., 2010; Gómez-Nicola et al., 2013; Walker et al., 2017). This proliferative activity is regulated by the tyrosine kinase, CSF1R; indeed, CSF1R-deficient mice and rats have no microglia (Ginhoux et al., 2010; Erblich et al., 2011; Gómez-Nicola et al., 2013; Pridans et al., 2018). Furthermore, CSF1R itself is upregulated in preclinical neurodegeneration models and human post-mortem samples (Akiyama et al., 1994; Murphy et al., 2000). Genetic and pharmacological strategies to eliminate or reduce CSF1R kinase activity, and thus decrease microglial proliferation, have been shown to promote beneficial effects in preclinical models of AD, ALS, tauopathies, and MS (Asai et al., 2015; Bruttger et al., 2015; Dagher et al., 2015; Borjini et al., 2016; Martínez-Muriana et al., 2016; Olmos-Alonso et al., 2016; Spangenberg et al., 2016, 2019; El-Gamal et al., 2018; Elmore et al., 2018; Sosna et al., 2018; Spiller et al., 2018; Mancuso et al., 2019; Zhong et al., 2019). Of note, a number of PET tracers have recently been developed to monitor response to CSF1R inhibitors which we believe reflects the broad level of interest in this mechanism (Tanzey et al., 2018; Horti et al., 2019; Janssen and Mach, 2019; Mason et al., 2019).

Colony stimulating factor 1 receptor has two known ligands, CSF1 (colony stimulating factor 1) which is also expressed in microglia and IL-34 predominantly expressed in neurons (Lin et al., 2008; Wei et al., 2010; Mizuno et al., 2011; Nandi et al., 2012; Wang et al., 2012; Chitu et al., 2016). Ligand binding triggers dimerization and transphosphorylation to activate the receptor and subsequently activate a downstream cascade of cytoplasmic mediators that enable development, survival or proliferation phenotypes (Akiyama et al., 1994; Xu et al., 2015; Askew et al., 2017). Mutations in CSF1R have been associated with adult-onset leukoencephalopathy with axonal spheroids and pigmented glia (ALSP) (Stabile et al., 2016; Lynch et al., 2017). Interestingly this condition can often be misdiagnosed as AD, corticobasal syndrome, DLB or atypical PD (Puschmann, 2013; Adams et al., 2018; Sharma et al., 2019). More recently, it has been reported that CSF1R may have variants associated with AD pathogenesis (Giau et al., 2019a, b).

#### Small Molecule Inhibitors

Structure-based drug development has been leveraged in some cases to rationally design CSF1R inhibitors including pexidartinib (PLX3397), GW2580, KI20227, AZD7507; JNJ-40346527, BLZ945/buparlisib or a set of DFG-out inhibitors (Ohno et al., 2006; Meegalla et al., 2008; Meyers et al., 2010; Uitdehaag et al., 2011; Pyonteck et al., 2013; Scott et al., 2013; Dagher et al., 2015). These molecules have largely been intended for indications such as rheumatoid arthritis and cancer related disorders where systemic exposure is required, thus these molecules have not been optimized for BBB penetration. Lack of selectivity is also a challenge with these molecules in CNS disorders; CSF1R is a class III receptor tyrosine kinase (RTK) which also includes cKIT, FLT3, and PDGFs in its family. The majority of CSF1R inhibitors also exhibit activity against these family members (as well as other kinases) and the converse is true, i.e., class III RTK inhibitors such as masitinib and sunitinib also inhibit CSF1R (Uitdehaag et al., 2011; Gandin et al., 2015). More recently, fragment-based drug design has been leveraged to identify novel starting points for CSF1R inhibition (Machiraju et al., 2019), presumably to better optimize selectivity and brain penetration.

#### Clinical Development

The broad-spectrum class III RTK inhibitor, masitinib, is currently in phase III for ALS (NCT02588677; NCT03127267) (Petrov et al., 2017; Mora et al., 2019) and has also been assessed in AD populations (NCT00976118), although detailed publications of these data are not yet available. Plexxikon initiated a Ph2a trial in AD populations in 2016 (Eudra 2016-000429-38) but no results have been published / reported to date.

#### Synopsis

While multiple data streams have indicated neuroinflammation and microglial activation as pathophysiological features of neurodegenerative disorders, it is not clear at what stage in the disease neuroinflammatory process would be the most appropriate point for intervention to lead to clinical benefit. There remains a question of when this detrimental effect of microglia may become relevant and consequently when would be an appropriate time to treat with this mechanism. The molecules developed and tested to date have suboptimal levels of CNS penetration and/or limited selectivity which again may impact the interpretation of the preclinical efficacy and ultimately clinical tolerability. That said there are preclinical data demonstrating a clear effect of CSF1R inhibitors (Olmos-Alonso et al., 2016; Mancuso et al., 2019; Spangenberg et al., 2019). The strong structural understanding of this kinase and significant interest in the target should prime the repurposing of existing molecules and the next generation of ligands designed for CNS indications.

### MAP3K12 (DLK, Dual Leucine Zipper Kinase)

#### Biological Rationale

Dual leucine zipper kinase, a mitogen-activated protein kinase kinase kinase, functions as an injury sensor that initiates the c-Jun N-terminal kinase (JNK)-dependent stress response in neurons to mediate context-dependent axon re- and degeneration (Gallo and Johnson, 2002; Hirai et al., 2006). DLK activation can lead to rapid neuronal degeneration while both the presence and absence of DLK has been reported to promote axon regeneration following injury (Chen et al., 2008; Hammarlund et al., 2009; Miller et al., 2009; Xiong et al., 2010; Ghosh et al., 2011; Itoh et al., 2011; Shin et al., 2012, 2019; Watkins et al., 2013; Welsbie et al., 2013; Wlaschin et al., 2018). The seemingly contradictory responses in neurons appear context dependent and the substrates for JNK (see section 3.6) phosphorylation differ accordingly (reviewed in Tedeschi and Bradke (2013) and Siu et al. (2018)]. In the context of chronic neurodegenerative disease, the experimental evidence (mostly from Genentech investigators), supports the notion that DLK is a conserved regulator of neuronal degeneration following injury. Aberrant activation of the DLK-JNK pathway has been reported in transgenic mouse models and spinal cord lysates from ALS patients (Le Pichon et al., 2017). Equivalent findings have been reported in two AD transgenic mouse models and in post-mortem CNS tissues from patients with disease (Le Pichon et al., 2017). Genetic manipulation or pharmacological inhibition of DLK, with compounds developed by Genentech, was reported to attenuate synaptic loss, neuronal degeneration and functional decline in models of AD, PD, and ALS in addition to acute neuronal injury models (Miller et al., 2009; Itoh et al., 2011; Pozniak et al., 2013; Watkins et al., 2013; Patel et al., 2015a, b, 2017; Le Pichon et al., 2017; Yin et al., 2017). It should be borne in mind that the closely related family member MAP3K13 (LZK, leucine zipper kinase) also regulates axonal responses to injury via JNK signaling, and notably, co-overexpression of both kinases did not have synergistic effects (Chen et al., 2016). The potential for complex functional interaction and cross-regulation should be considered in the context of therapeutic development.

#### Small Molecule Inhibitors

Small molecule DLK inhibitors that reduced c-Jun phosphorylation in mouse models were initially identified following high throughput screening approaches and refined using a shape-based scaffold hopping approach (Patel et al., 2015a, b). Structure-based design was leveraged to further optimize two series with respect to pharmacokinetics (PK, particularly brain penetrance), selectivity and tolerability profiles with potential for prolonged administration exemplified by GDC-0134 (Patel et al., 2017). The selectivity profile indicates 23-fold selectivity against the closely related family member MAP3K13; which may functionally compensate in some contexts. Other molecules such as tozasertib or sunitinib have been reported to have DLK inhibitory activity, based on high content screening using retinal ganglion cells, but have not been optimized for selectivity or PK parameters commensurate with CNS exposure (Welsbie et al., 2013, 2017). More recently, a platform capable of identifying inhibitors of DLK palmitoylation in order to modulate kinase localization has been reported (Martin et al., 2019).

#### Clinical Development

Genentech’s DLK inhibitor molecule is currently in PhI for ALS (NCT02655614).

#### Synopsis

This case study represents a clear example of how structural and medicinal chemistry know how has been applied to actively develop a kinase inhibitor with CNS penetrance. It remains to be seen whether the preclinical assessment was sufficient to mitigate the risks associated with apparent context-dependent effects of DLK inhibition as the published data may suggest a narrow therapeutic index with potential toxicology concerns.

### GSK3B (Glycogen Synthase Kinase 3β, GSK3β)

#### Biological Rationale

*GSK3B* codes for two isoforms of a constitutively active serine/threonine kinase which phosphorylates over 40 substrates with key roles in Wnt signaling, insulin signaling, glycogen metabolism, development, mitotic regulation, inflammation (particularly the innate immune response), neurotransmitter and neurotrophic factor signaling and microtubule dynamics (Rayasam et al., 2009; Patel and Woodgett, 2017). GSK3β activity is tightly regulated by several mechanisms including cellular localization, protein interactions and phosphorylation, leading to increasing (Y216 in the activation loop) or decreasing (S9) substrate binding ability (Hoshi et al., 1996; Haar et al., 2001; Bijur and Jope, 2003; Dajani et al., 2003). Increased expression and activity of GSK3β has been noted in AD, PD, and ALS post-mortem tissue while decreased expression and activity was reported in Huntington’s disease (HD) patient brains (Pajak et al., 2009; Lim et al., 2014; Credle et al., 2015; Nicolia et al., 2017). Furthermore, rare variants have been identified as causative in AD and FTD (frontotemporal dementia) (Schaffer et al., 2008; Kettunen et al., 2015). Treatment with GSK3β inhibitors has been shown to be impactful in a number of models, but there have also been challenges given the toxicities associated with some compounds (Selenica et al., 2007; Peineau et al., 2009; Petit-Paitel et al., 2009; Serenó et al., 2009; Eldar-Finkelman and Martinez, 2011; Ahn et al., 2012; Kramer et al., 2012; Fuchs et al., 2015). GSK3β was identified as a tau kinase in addition to directly promoting Aβ production in AD pathophysiology (Hanger et al., 1992; Sperbera et al., 1995; Spittaels et al., 2000; Phiel et al., 2003; Terwel et al., 2008). Accordingly, some inhibitors have been shown to reverse tau hyperphosphorylation and subsequently resulted in some beneficial outcomes in models (Martinez et al., 2002; Serenó et al., 2009; Onishi et al., 2011; Berg et al., 2012; Kramer et al., 2012; Xiong et al., 2013).

#### Small Molecule Inhibitors

Phosphorylation of target proteins by GSK3β generally inhibits their activity; inhibitor treatment thus disinhibits the pathway(s) in question. Analysis of the GSK3β structure as an unphosphorylated apo-enzyme together with Y216 monophosphorylated enzyme complexed with a peptide provided insight into both its regulation of the kinase activity and its preference for pre-phosphorylated substrates (Bax et al., 2001; Dajani et al., 2001, 2003; Haar et al., 2001). Diverse GSK3β inhibitors have been developed and can be grouped according to their mechanism of action and binding affinity: (1) ATP-competitive inhibitors which constitute the largest pool with a diverse range of scaffolds such as SB-216763 and AZD1080 (Georgievska et al., 2013; Li et al., 2015). These and other ATP-competitive inhibitors have been shown to exhibit BBB penetrance including pyrazines or oxadiazoles (Saitoh et al., 2009; Onishi et al., 2011; Berg et al., 2012; Yngve et al., 2012; Pandey and DeGrado, 2016; Prabhakaran et al., 2017). (2) non-ATP competitive inhibitors which either inhibit by binding at a catalytic triad and resisting the correct substrate orientation, such as tideglusib (also known as NP-12) and a thiadiazolidinone derivative (which has shown evidence of brain penetration) (Luna-Medina et al., 2007; Pandey and DeGrado, 2016), or by directly competing with the substrate; (3) irreversible inhibitors (Perez et al., 2009); (4) peptide-like inhibitors that act as pseudo-substrates; (5) allosteric inhibitors which bind in non-ATP and non-substrate competitive fashion and thus have potential to enable a more selective and subtle modulation of the enzyme (Martinez et al., 2002; Palomo et al., 2011, 2017) and (6) metal ions such as lithium chloride which has not been included in this review, given that it is neither specific nor potent (Maqbool et al., 2016). Structural analyses of chemically diverse inhibitors in complex with GSK3β have illuminated how the enzyme interacts with selective and non-selective inhibitor scaffolds and have provided insight and opportunity to guide further rational design approaches (Bertrand et al., 2003; Kramer et al., 2012; Sivaprakasam et al., 2015).

#### Clinical Development

Despite extensive promising preclinical data, GSK3β inhibitors have rarely reached clinical trials (Pandey and DeGrado, 2016). Molecules that have reached human testing for neurodegenerative indications are tideglusib (NCT00948259; NCT01049399; NCT01350362) and AZD1080. No clinical pharmacokinetic data for tideglusib has been reported despite a radiolabeled analog being reported (Pandey and DeGrado, 2016). Early studies in AD patients suggested decreased brain atrophy, reduction of phospho-tau and BACE-1 levels in cerebrospinal fluid and improved cognitive function (del Ser et al., 2013). Further assessment of tideglusib suggested no clinical benefit although it was well tolerated (up to a 1000 mg qd for 24 weeks) (Lovestone et al., 2015). Tideglusib treatment was additionally reported to decrease brain atrophy in progressive supranuclear palsy (PSP) following 1 year of treatment with 600 or 800 mg daily but with no other clinical benefits (Höglinger et al., 2014; Tolosa et al., 2014). In Phase I trials, AZD1080 was apparently safe and well tolerated with reasonable PK profiles and evidence of peripheral target engagement (Georgievska et al., 2013). No further development has been published since. ALS was also being evaluated as a potential indication (MR/K015273/1) but again no clinical progression has been reported.

#### Synopsis

Published data suggests GSK3β inhibition may have therapeutic benefit across a range of neurodegenerative diseases. Many small molecule inhibitors have been generated, of which some appear to have a reasonable degree of CNS penetration. Toxicity may be one potential issue, likely due in part to the essential role of the enzyme in many fundamental physiological pathways, which has limited or prevented further exploration. Nonetheless, two molecules have been identified as being sufficiently safe for clinical investigation but have not yielded positive data for CNS indications.

### c-Jun N-Terminal Kinases (JNKs, Particularly JNK3/*MAPK10*)

#### Biological Rationale

c-Jun N-terminal kinases are members of the mitogen-activated protein kinase (MAPK) family and regulate serine/threonine phosphorylation of several transcription factors in response to various stimuli, e.g., endoplasmic reticulum stress / unfolded protein response signaling, oxidative and excitotoxic stress, DNA damage, neurotoxins, cytokines, and fatty acids (Haeusgen et al., 2009; Bogoyevitch et al., 2010). Activation of the JNK pathway, through phosphorylation in the activation loop by MAP2Ks, relies on the coordinated interaction of scaffold proteins enabling the assembly of signaling complexes and recruit JNK-phosphorylated transcription factors (Mooney and Whitmarsh, 2004; Guo and Whitmarsh, 2008; Engström et al., 2010; Antoniou et al., 2011; Zhan et al., 2015, 2016). JNKs have been implicated in a range of neurodegenerative disorders including PD and AD (Chambers and LoGrasso, 2011; Crocker et al., 2011; Mehan et al., 2011; Yarza et al., 2016). Increased JNK phosphorylation has been reported in human post-mortem AD patient brains; together with reports of tau and amyloid precursor protein (APP) phosphorylation resulting in a stimulation of Aβ42 peptide production (Yoshida et al., 2004; Kimberly et al., 2005; Lagalwar et al., 2006; Pearson et al., 2006; Vogel et al., 2009; Killick et al., 2014; Gourmaud et al., 2015). Genetic depletion and pharmacological inhibition of JNK have been found impactful in AD *in vivo* model phenotypes (Bogoyevitch et al., 2004; Bowers et al., 2011; Probst et al., 2011; Ramin et al., 2011; Hong et al., 2012; Zhou et al., 2015; Petrov et al., 2016; Gourmaud et al., 2018). Increased nuclear staining of c-Jun (JNK substrate) as a surrogate marker for JNK activity has been observed in patients with PD but not in dementia with Lewy bodies (DLB) (Ferrer et al., 2001; Hunot et al., 2004). JNK3, the predominant CNS expressed isoform with roles in brain development as well as learning and memory function (Kuan et al., 1999; Bevilaqua et al., 2003; Brecht et al., 2005; Waetzig et al., 2006; Eminel et al., 2008). Kuan et al. (1999), Bevilaqua et al. (2003), Brecht et al. (2005), Waetzig et al. (2006) and Eminel et al. (2008) is robustly activated in toxin models of PD while genetic deletion of JNK3 and pan-kinase inhibition are both protective in these models. However, protective effects of JNK3 inhibition / deletion in genetic models of PD are less clear (Choi et al., 1999, 2015; Saporito et al., 1999, 2000; Hunot et al., 2004; Wang et al., 2009; Chambers et al., 2011, 2013). Complementary data exists for other neurodegenerative disease models with respect to genetic and pharmacological interrogation (Morfini et al., 2009; Perrin et al., 2009; Suzuki and Matsuoka, 2013; Wityak et al., 2015). Finally, JNK3 has also been identified as a genetic modifier for spinal and muscular atrophy (Genabai et al., 2015).

The JNK-interacting protein 1 (JIP1) derived peptide inhibitor (D-JNK1-1, also known as XG-102, AM-111 or brimapitide) has the advantage of comparative specificity for JNK and is fused to the Tat peptide sequence to enable cellular delivery across the BBB (Barr et al., 2002; Davoli et al., 2014). Peptide treatment has been shown to be efficacious in multiple *in vivo* models (Repici et al., 2008; Colombo et al., 2009; Sclip et al., 2011, 2014; Tran et al., 2012). More recent preclinical data suggests that peptide inhibitor treatment in the 5xFAD model improves cognitive performance and decreases amyloid load, mirroring the effect of genetic deletion of JNK3 (Gourmaud et al., 2018). In addition, several preclinical studies revealed a protective effect of the peptide inhibitors in hearing loss following acoustic trauma; this led to subsequent clinical development for acoustic trauma and acute sensorineural hearing loss resulting in statistically significant positive effects (Grindal et al., 2010; Omotehara et al., 2011; Suckfuell et al., 2014; Eshraghi et al., 2018).

#### Inhibitors

Several ATP-competitive JNK inhibitors have been developed with a range of characteristics including good CNS penetration; only three are mentioned here as illustrations of their class. AS602801 (bentamapimod) is a pan-JNK inhibitor with good bioavailability and able to cross the BBB (Ferrandi et al., 2011). SP600125 is a CNS penetrant and reversible anthrapyrazolone ATP competitive inhibitor, 300-fold more selective for JNK versus ERK and p38 MAPK (Bennett et al., 2001; Vogel et al., 2009; Davies and Tournier, 2012). CC-930 (tanzisertib) is a potent, selective, orally active JNK inhibitor selective against ERK1 and p38α (Krenitsky et al., 2012). More recently, J30-8 was reported to have over 2500-fold isoform selectivity for JNK3, which could make it a good tool molecule for further mechanistic studies in ND models (Dou et al., 2019).

A structural class analysis of over 40 JNK structures, both *apo* and in complex with ligands or peptides has suggested the possibility for allosteric interactions between the catalytic and peptide binding sites and the A-loop (Ansideri et al., 2018). This has revealed two potential distinct autoinhibitory mechanisms leading to blocking of the formation of the active catalytic site in the inactive kinase (Laughlin et al., 2012; Mishra and Günther, 2018). Furthermore, structural assessments of peptide binding modes indicate the basis for selectivity of different docking motifs.

#### Clinical Development

A few small molecule JNK inhibitors have entered clinical trials including tanzisertib and bentamapimod but none for the treatment of neurodegenerative disorders. While tanzisertib appeared safe and well-tolerated, observations of dose-responsive decreases in MMP7 plasma levels halted clinical development. Clinical trials with molecules further upstream of the cascade have also been reported, such as CEP-1347, a mixed lineage kinase inhibitor, which failed to show benefit in PD patients (Shoulson and Parkinson’s Study Group and Precept Investigators, 2006; Parkinson Study Group, 2007). It is worth noting that DLK (discussed above) is also upstream in the JNK cascade so may ultimately provide evidence to indicate JNK utility in ALS if the Genentech molecule continues to progress.

#### Synopsis

The JNK cascade appears to be a relevant axis with rationale for inhibitor development in both AD and PD. While there has been progress in inhibitor design, the specificity across JNK isoforms, in addition to other kinases such as MAPK14, with a brain penetrant small molecule is still a challenge that has not yet been appropriately overcome. Improved selectivity as a key strategy is likely only achievable via targeting interactions such as JIP1 outside of the ATP pocket.

### LRRK2 (Leucine Rich Repeat Kinase 2)

#### Biological Rationale

Leucine rich repeat kinase 2 is a large, multi-domain enzyme with dual GTPase and kinase activities that has been genetically linked to sporadic and familial PD (Zimprich et al., 2004; Khan et al., 2005; Ross et al., 2011; Paisán-Ruiz et al., 2013). Mutations are distributed throughout the protein, predominantly in the kinase and ROC/GTPase domain and have been shown to modulate ATP or GTP hydrolysis rates *in vitro* (Smith et al., 2005; West et al., 2005; Gloeckner et al., 2006; Greggio et al., 2006; Nichols et al., 2010; Dusonchet et al., 2011; Cookson, 2015; Manzoni et al., 2015). Some of these data implicate increased kinase activity (and resultant phosphorylation of downstream Rab proteins) with specific mutations such as G2019S, which has spurred development of LRRK2 kinase site inhibitors. It is noteworthy that other mutations have not recapitulated these observations (Taylor and Alessi, 2020). A computational model of the full length homodimeric protein supports the notion of cross-regulation between the GTPase and kinase domains and suggest that downstream biochemistry may be more complicated than a simple enhancement of kinase activity (Mata et al., 2006; Greggio et al., 2008; Weiss, 2008; Berger et al., 2010; Guaitoli et al., 2016; Rosenbusch and Kortholt, 2016). In particular, modeling approaches have also enabled predictions of repeat domain structure and impact of mutations using other organism homologs and the overall view is one of a compact architecture with tight, multidomain organization and intramolecular regulation of the enzymatic activities (Vancraenenbroeck et al., 2012; Mills et al., 2014; Guaitoli et al., 2016). Crystal structures for the LRRK2 ROC (Ras of complex proteins) domain in complex with GDP-Mg^2+^ further support the notion that LRRK2 may actually function as a dimeric GTPase (Deng et al., 2008; Deyaert et al., 2017; Nguyen and Moore, 2017; Mills et al., 2018).

Leucine rich repeat kinase 2 is regulated by autophosphorylation and phosphorylation by other kinases and interactions with kinase-specific chaperones (Chia et al., 2014; Muda et al., 2014; Shu et al., 2016). Unbiased protein-protein interaction and other approaches have provided insights into the functional roles of LRRK2 including the regulation of vesicular and lysosomal trafficking, lysosomal maturation and autophagy regulation (Shin et al., 2008; Tong et al., 2012; Manzoni et al., 2013, 2016; Beilina et al., 2014; Cirnaru et al., 2014; Dodson et al., 2014; Gómez-Suaga et al., 2014; Parisiadou et al., 2014; Piccoli et al., 2014; Saez-Atienzar et al., 2014; Schapansky et al., 2014; Stafa et al., 2014; Waschbüsch et al., 2014; Yun et al., 2015; Bang et al., 2016; Islam et al., 2016; Kuwahara et al., 2016; Roosen and Cookson, 2016; Steger et al., 2016; Hur et al., 2019; Lewis, 2019). In support of this genetic ablation of LRRK2 leads to reduced autophagic clearance and accordingly, α-synuclein accumulation in the absence of neurodegeneration (Biskup et al., 2006; Giasson et al., 2006; Higashi et al., 2007; Alegre-Abarrategui et al., 2009; Tong et al., 2012; Baptista et al., 2013; Ness et al., 2013; Longo et al., 2017). It is noteworthy that although LRRK2 is expressed in various brain regions it also has broad systemic expression across several other organs such as lung, kidney, and heart.

#### Inhibitors

Several molecules were assessed and/or developed to inhibit LRRK2 kinase function based on the observations of increased kinase activity and associated neurotoxicity with the most common mutation, G2019S [reviewed in Zhao and Dzamko (2019)]. Of note, compounds have been developed specifically with the aims of combining selectivity and brain penetrance. An HTS and subsequent lead optimization provided the low nM reversible ATP competitive inhibitor LRRK2-IN-1 but PK studies revealed limited BBB permeation (Deng et al., 2011). The anthracene and phenanthrene derivatives LDN-73794 and LDN-22684 inhibited LRRK2-G2019S and wild-type LRRK2 in the low micromolar range but with different mechanisms of inhibition. LDN-73794 was confirmed to be ATP competitive, whereas LDN-22684 was found to be a non-ATP-competitive inhibitor and furthermore was not GTP- nor substrate-competitive and was thus deduced to be an allosteric inhibitor (Liu et al., 2010a, b). GSK2578215A was another highly potent and selective LRRK2 inhibitor with high brain exposures but unfortunately failed to inhibit LRRK2 in mice, potentially due to species differences (Reith et al., 2012). The initial HTS output GNE-7915 was further developed to identify the analogs GNE-0877 and GNE-9605; all of which were potent LRRK2 inhibitors with enhanced brain penetration and robust, dose-dependent inhibition in mice and non-human primates (NHP) (Estrada et al., 2014; Fuji et al., 2015). PF-06447475 is another second-generation molecule with high potency, selectivity and good BBB permeability properties but poor oral PK profiles (Daher et al., 2015; Henderson et al., 2015). Finally, an indazole termed Mli-2 was reported to exhibit a marked improvement in potency for G2019S LRRK2, orally available with good brain penetrance/PK profiles and inhibitory activities (Fell et al., 2015; Scott et al., 2017). For completeness, we also acknowledge here the development of GTP binding inhibitors which have the potential of impacting on kinase function through interaction of the two domains, although these molecules are considerably behind the plethora of kinase domain inhibitors (Sen et al., 2009; Li et al., 2014, 2015). Alternatively, the structural interface between LRRK2 and the 14-3-3 protein, a key interaction partner for LRRK2, has been reported (Stevers et al., 2017, 2018). Many pathogenic mutations in LRRK2 disrupt or weaken the dimeric or the 14-3-3 interactions; suggesting a stabilization approach as a potential alternative therapeutic strategy (Nichols et al., 2010; Fraser et al., 2013; Melrose, 2015; Zhao et al., 2015; Lavalley et al., 2016).

#### Clinical Development

Preclinical safety testing for LRRK2 inhibitors has been challenging, partially due to robust expression of LRRK2 in the lung, kidney and some peripheral immune cells. Knockout mice have a normal life span, but pathology has been noted in peripheral organs, particularly the kidney (Herzig et al., 2011; Tong et al., 2012; Ness et al., 2013). Kidney phenotypes have not been observed in rodents or NHP treated with LRRK2 inhibitors (Fell et al., 2015). However, phospholipidosis and various pathologies have been observed in lungs of NHP with several structurally diverse inhibitors, which appeared to be reversible upon withdrawal of the compounds, together suggesting on-target toxicity (Herzig et al., 2011; Baptista et al., 2013, 2020; Ness et al., 2013; Shayman and Abe, 2013; Daher et al., 2015; Fuji et al., 2015). Interestingly, chronic pharmacological inhibition of LRRK2 appears to result in reduced expression of the protein by promoting its ubiquitination and consequent proteasomal degradation (Herzig et al., 2011; Fuji et al., 2015; Zhao et al., 2015; Lobbestael et al., 2016; Perera et al., 2016). In this context, it will be interesting to ascertain effects of antisense oligonucleotides such as BIIB094 against specific mutations in LRRK2 (NCT03976349). The Genentech compounds have been licensed to Denali and are currently in the clinic for Phase 1b trials as DNL151 and DNL201 (NCT04056689 and NCT03710707). Denali press releases have since reported both DNL151 and 201 are well-tolerated with no obvious functional evidence of lung toxicology, and additionally have high levels of target and pathway engagement with modulation of lysosomal biomarkers^3^.

#### Synopsis

The genetic linkage in LRRK2 to PD susceptibility together with altered protein activity provides the rationale for inhibitor development in PD. There are now several chemically diverse, potent, selective and brain penetrant inhibitors. However, the apparent on-target toxicity seen with some of these inhibitors in NHP raises concerns around the therapeutic index and safety of chronic dosing which have impeded clinical investigations with many of these molecules. The ongoing trials are seeking to leverage both safety and efficacy biomarkers with a view to impacting Parkinson’s onset/progression in a genetically stratifiable cohort.

### MAPK14 (p38α Mitogen Activated Protein Kinase)

#### Biological Rationale

P38α is a ubiquitously expressed serine/threonine kinase activated by upstream MAP2Ks in response to environmental and inflammatory/oxidative/metabolic/ER stress. In the CNS, p38α (like JNK3) has been proposed to mediate synaptic function, proteostasis and endolysosomal function in response to stress signals and indeed, it is likely that there is cross-regulation between the p38 and JNK pathways [reviewed in Borders et al. (2008)]. Accordingly, the MAPK stressor signal cascade has broad literature representation in neurodegenerative disease, and p38α is no exception with specific dysregulation observed at early stages in AD, ALS, PD, FTD, and PSP (Hensley et al., 1999; Atzori et al., 2001; Hartzler et al., 2002; Raoul et al., 2002; Sun et al., 2003). P38α, like GSK3β or CDK5-p25, has been hypothesized to link Aβ and tau or α-synuclein (Puig et al., 2004; Grassi et al., 2019). Genetic suppression of p38α has been reported to be beneficial in AD models and pharmacological inhibition of p38α has been claimed to confer benefits *in vivo* (Alam, 2015; Roy et al., 2015; Maphis et al., 2016; Schnöder et al., 2016; Colié et al., 2017; Lee and Kim, 2017; Zhou et al., 2017).

#### Small Molecule Inhibitors

While potency, selectivity and SAR data is available for several individual p38α inhibitors; Astolfi and colleagues were the first to systematically assess these from a structural perspective [reviewed in Astolfi et al. (2018)]. Both ATP-competitive and non-competitive molecules have been identified such as VX-745 (neflamapimod), MW150 and the related MW181, PH-797804, BMS-582949, PF-03715455 and an allosteric binder. Notably, multiple structures have been generated with both ATP- and non-ATP site binders, often through an attempt to improve on selectivity profiles (Fitzgerald et al., 2003; Swahn et al., 2005; Sack et al., 2008; Scior et al., 2011; Arai et al., 2012; Getlik et al., 2012; Astolfi et al., 2019) and reviewed in Astolfi et al. (2018).

#### Clinical Development

Neflamapimod (VX745) was discovered through structure-based design at Vertex and previously assessed in rheumatoid arthritis in 2001. The molecule appears to be tolerated up to 125 mg twice daily for 12 weeks (Alam et al., 2017). As a result of these data and the preclinical profile, a Phase IIa study in patients with early stage AD (6–12 weeks in patients with MMSE 20-28 and biomarker positive) was performed and demonstrated that neflamapimod treatment led to significant improvement in episodic memory (Alam et al., 2017). A blinded, placebo-controlled Phase 2 study of 152 people with CSF biomarker-confirmed mild AD was initiated in December 2017, with clinical output expected late 2019 (NCT03402659). Additional clinical studies of neflamapimod in DLB (NCT04001517) and HD (NCT03980938) have since been initiated.

#### Synopsis

The notion of MAPK pathway involvement in neurodegenerative disease is broadly supported in the literature. However, targeting these pathways are challenging due to (1) “crossover” between parallel pathways and (2) achieving selectivity and sufficient CNS exposure without notable systemic levels for a small molecule against any kinase in this pathway, including p38α. No drugs targeting this kinase are on the market yet for any indication, including chronic obstructive pulmonary disease, rheumatoid arthritis and corticosteroid-resistant asthma. Indeed, more than a dozen distinct molecules have failed in the clinic at least partly due to hepatotoxicity and development of tolerance. However, the reported favorable profile of neflamapimod and multiple ongoing clinical studies will hopefully inform whether this mechanism has therapeutic benefit.

### MTOR (Mechanistic Target of Rapamycin Complex 1, mTOR/mTORC1)

#### Biological Rationale

The serine/threonine kinase MTOR is a regulatory nexus for cell growth and metabolism in response to nutrients, growth factors and cellular energy conditions modulating autophagy, glucose metabolism, protein synthesis, and mitochondrial functions. MTOR is the catalytic subunit of two structurally and functionally distinct complexes with respect to protein composition: the rapamycin sensitive mTORC1 which functions as a nutrient sensor activated by insulin, growth factors and amino acids; and mTORC2 which has a role in cytoskeleton organization. Under nutrient-replete conditions, mTORC1 as an autophagy repressor via phosphorylation of ULK1, ATG13, ATG14L, and TFEB, key positive regulators of autophagy; and a positive regulator of protein synthesis via phosphorylation of S6K and eIF4E BP1-4 thus promoting global and cap-end translation (Rabanal-Ruiz et al., 2017; Walters and Cox, 2018). More recently, cryoEM data have refined our thinking as to how MTOR interacts with and is regulated by mTORC1 complex members such as LST8, LAMTOR (“Ragulator”), Raptor, Rheb, PRAS40, and chaperonins (Yang et al., 2016; Yang H. et al., 2017; de Araujo et al., 2017; Cuéllar et al., 2019).

A consistent observation across neurodegenerative disease pathophysiology is the occurrence of specific misfolded proteins. These proteins ultimately appear as aggregates/inclusions that are proposed to infringe on normal cellular function including impaired protein clearance either by the ubiquitin-proteasome pathway or the lysosomal degradation pathway (including autophagy) [reviewed in Frake et al. (2015) and Menzies et al. (2017)]. Several studies have implicated impaired autophagy and aberrant mTOR signaling in models and/or post-mortem brain samples of various neurodegenerative diseases (Webb et al., 2003; Li et al., 2005; Nixon et al., 2005; Yu et al., 2005; Boland et al., 2008; Pickford et al., 2008; Jaeger and Wyss-Coray, 2009; Chu, 2010; Dehay et al., 2010; Heng et al., 2010; Spilman et al., 2010; Winslow et al., 2010; Zheng et al., 2010; Bové et al., 2011; Caccamo et al., 2011; Barmada et al., 2014; Manzoni et al., 2016). A relevant therapeutic approach could thus include facilitating the clearance of aberrantly folded proteins by increasing autophagy through repressing mTORC1; consistent with this notion, rapamycin treatment restores mTOR activity and rescues neurodegenerative disease model phenotypes (Webb et al., 2003; Ravikumar et al., 2004; Sarkar et al., 2009; Dehay et al., 2010; Menzies et al., 2010; Spilman et al., 2010; Bové et al., 2011; Cortes et al., 2012; Ozcelik et al., 2013; Jiang et al., 2014). It is also noteworthy that this approach will have effects beyond the CNS as illustrated in SOD1 mouse models for ALS, where both detrimental and beneficial immunomodulatory effects have been reported (Zhang et al., 2011; Staats et al., 2013).

#### Small Molecule Inhibitors

The allosteric mTORC1 inhibitor rapamycin (also known as sirolimus) was the first drug to be identified as an autophagy inducer through its interaction with FKBP12 which then binds to and inhibits the kinase activity of mTORC1 (Vézina et al., 1975; Heitman et al., 1991; Van Duyne et al., 1993; Choi et al., 1996). Crystal and cryoEM structures have illustrated how the FKBP12-rapamycin binding domain appears to regulate access to the active site and thus blocks substrate recruitment preventing downstream phosphorylation of regulatory proteins such as 4E-BP1 and S6K1 (Choi et al., 1996; Yip et al., 2010; Yang et al., 2013, 2016). Rapamycin together with other related natural product “rapalogs” such as temsirolimus (CCI-779), everolimus (RAD001), and ridaforolimus (AP23573) have good selectivity for mTORC1 versus mTORC2 but suboptimal exposure levels in the brain (O’Donnell et al., 2008; Benjamin et al., 2011). ATP-competitive inhibitors of mTOR inhibitors such as Torin 1, WYE-125132, and AZD-8055 have lower selectivity profiles for mTORC1 versus mTORC2 due to its mode of action and accordingly are able to block phosphorylation of all substrates of the two complexes as opposed to a subset of substrates. These ATP-competitive inhibitors are viewed as being more promising in oncology for tumors that are addicted to the mTOR signaling pathway and in line with this, suboptimal levels of BBB penetration in addition to toxicity issues with chronic dosing have been reported (Thoreen et al., 2009; Yu et al., 2010; Jhanwar-Uniyal et al., 2019).

#### Clinical Development

Rapamycin and its analogs have been used for many years in organ transplant patients (for review see Waldner et al., 2016) but its suggested utility in CNS disorders has been a more recent development driven by preclinical evidence of efficacy (reviewed above). These preclinical data together with the good safety profile led to the initiation of a phase II clinical assessment of rapamycin in an ALS population (NCT03359538). Unfortunately, no clinical outcome data has been published to date and no other trials of rapamycin or its analogs have been reported. Of note, everolimus appears to have better oral bioavailability and partitioning to the CNS and thus may be an alternative option for clinical development although none has been instigated to date for neurodegenerative indications (Frost and Hulbert, 2015; Palavra et al., 2017).

#### Synopsis

The notion of augmenting autophagic flux for neurodegenerative disease is cogent and warrants detailed assessment. Indeed, many organizations are pursuing approaches to do just that. Consistent with the hypothesis, inhibition of mTORC1 has consistently been beneficial in multiple neurodegenerative disease models but clinical confirmation is still awaited. This is likely because current ATP competitive inhibitors are suboptimal with respect to selectivity profile, tolerability and CNS exposure levels. Allosteric inhibitors with improved selectivity for mTORC1 may have benefit but we suggest that further improvement of these molecules or alternative approaches to impacting mTORC1 complex such as via regulators of the complex may have greater therapeutic potential.

### RIPK1 (Receptor-Interacting Serine/Threonine-Protein Kinase 1)

#### Biological Rationale

Receptor-interacting serine/threonine-protein kinase 1 functions in a variety of pathways related to both cell survival and death. It has been linked to lysosome function (via cystatin F), inflammation (via p65 and cFos) and cell death pathways (apoptosis and necroptosis but not necrosis) (Wegner et al., 2017). For necroptosis, RIPK1’s kinase function appears essential, and it is a key part of a multiprotein complex termed the “necrosome” which triggers pathways such as the activation, phosphorylation and oligomerization of MLKL (Vandenabeele et al., 2010; Oberst, 2016). Inhibition of necroptosis has been suggested as a therapeutic strategy in diseases such as ALS and AD where RIPK1 activation has been reported (Li et al., 2012; Kaiser et al., 2014; Re et al., 2014; Vanden Berghe et al., 2015; Ito et al., 2016; Caccamo et al., 2017; Ofengeim et al., 2017; Degterev et al., 2019). However, it is not yet clear whether necroptosis is one of the key mechanisms by which neurons die (Chi et al., 2018; Heckmann et al., 2019) or if blocking it would be beneficial in the long run. Studies with optineurin knockout mice, and TBK1-TAK1 heterozygous KO mice, demonstrated RIPK1 activation [reviewed in Markovinovic et al. (2017)]. Targeting RIPK1 kinase function mitigated the axon-myelin phenotype and axonal loss observed in these models (Re et al., 2014; Ito et al., 2016; Markovinovic et al., 2017; Xu et al., 2018). Additionally, the RIPK1 inhibitor, necrostatin (Nec-1s), has been reported to be protective in the R6/2 transgenic mouse model of HD in response to ischemic insults *in vivo* (Zhu et al., 2011). Similarly, treatment of with Nec-1s has been reported to exert a protective effect in ALS and PD models (Re et al., 2014; Wu et al., 2015; Lin et al., 2019; Machado et al., 2019).

#### Small Molecule Inhibitors

Multiple small molecule inhibitors for RIPK1 inhibition have been developed with a range of binding modes. Allosteric binders such as Nec-1s are precedented in addition to ATP competitive molecules such as GSK2982772 (Najjar et al., 2015; Harris et al., 2017; Hamilton et al., 2019). The structural basis for kinase-domain based inhibition of RIPK1 by Nec-1s is reasonably well understood whereby the molecule stabilizes the protein in an inactive conformation through interactions between key amino acids in the activation loop and the surrounding structural elements; echoing the mechanism seen with ABL1 inhibitors (Xie et al., 2013; Najjar et al., 2015; Degterev et al., 2019). More recently, the trend has been to leverage structural information to guide inhibitor discovery toward type 1 and type 2 binding modes (Najjar et al., 2015; Harris et al., 2017; Hofmans et al., 2018). Interestingly, a number of kinase inhibitors defined against other kinase targets including tozasertib/VX-680, sorafenib, ponatinib and the PERK inhibitors GSK2606414/GSK2656157 have all been shown to have notable activity against RIPK1 (Fauster et al., 2015; Martens et al., 2017; Rojas-Rivera et al., 2017; Hofmans et al., 2018). There is little information available about DNL747 although DNL104, an earlier generation RIPK1 inhibitor licensed from the Yuan lab by Denali, may be related to necrostatins (Degterev et al., 2008; Wu et al., 2013).

#### Clinical Development

Denali has announced that they have completed recruitment for assessment of DNL747, a claimed potent, oral dosed brain penetrant RIPK1 inhibitor, in ALS (NCT03757351) and AD (NCT03757325) populations. These studies should read out in mid-2020. Another Denali molecule, DNL104, a BBB-penetrant RIPK1 inhibitor has been reported to modulate pharmacodynamic markers in a healthy volunteer trial (Grievink et al., 2019). GSK2982772 has been shown to be safe, tolerated, with good target engagement and is currently in Ph2a for chronic inflammatory diseases such as ulcerative colitis and psoriasis; but CNS penetrance has not been reported (NCT02776033, NCT02858492) (Weisel et al., 2017).

#### Synopsis

Although the rationale for this mechanism in necroptosis pathways seems reasonable, the clear weakness is the lack of clarity around the role this process plays in ongoing neurodegeneration; and whether blocking what is presumably quite a late stage cell death process will have real beneficial therapeutic consequences to the patient.

### ROCK (Rho-Associated Protein Kinase)

#### Biological Rationale

Actin is one of three key components of the neuronal cytoskeleton and contributes to maintaining the axonal and somatodendritic compartments: the others being intermediate filaments and microtubules. A clear connection between cytoskeleton damage and neurodegeneration has emerged over the past few years such as actin cytoskeleton dysregulation and axonal transport defects [microtubule dependent and independent; reviewed in Eira et al. (2016) and Kounakis and Tavernarakis (2019)]. The actin cytoskeleton is regulated by Rho GTPase family signaling pathways (which are themselves dysregulated in PD, HD, AD, and ALS) via ROCK (Minamide et al., 2000; Maloney et al., 2005; Fulga et al., 2007; Mendoza-Naranjo et al., 2007; Petratos et al., 2008; Munsie and Truant, 2012; Tourette et al., 2014). ROCK1 and 2 have overlapping substrates outside of cytoskeletal proteins including those related to autophagy and vesicle dynamics (reviewed in Koch et al., 2018). ROCK1 and 2 are normally present in an autoinhibited configuration but over-activation has been implicated in AD, PD, HD, and ALS (Petratos et al., 2008; Barcia et al., 2012; Villar-Cheda et al., 2012; Herskowitz et al., 2013; Conti et al., 2014; Fujita and Yamashita, 2014; Tönges et al., 2014; Eira et al., 2016; Gentry et al., 2016; Henderson et al., 2016; Narayanan et al., 2016; Saal et al., 2017). More recently, it has been reported that ROCK inhibitors upregulate the neuroprotective Parkin-mediated mitophagy pathway (Moskal et al., 2020). Genetic or pharmacological approaches using ROCK inhibitors such as fasudil and/or Y-27632 were beneficial in multiple disease models including ALS models which may be linked with the profilin genetic association in some forms of familial ALS (Zhou et al., 2003; Shao et al., 2008; Bauer et al., 2009; Deyts et al., 2009; Li et al., 2012; Tönges et al., 2012, 2014; Takata et al., 2013; Günther et al., 2014; Tatenhorst et al., 2014; Gentry et al., 2016; Saal et al., 2017). In addition to canonical ROCK signaling, it has been reported that ROCK activity plays a role in microglial phenotypes whereby activity is required to maintain proinflammatory (“M1”) phenotypes and ROCK inhibition enables a switch to an anti-inflammatory (“M2”) behavior (Zhang et al., 2013; Roser et al., 2017; Scheiblich and Bicker, 2017).

#### Small Molecule Inhibitors

Several ROCK inhibitors have been developed in the past few decades that are predominantly ATP-competitive type 1 inhibitors exemplified by fasudil, the closely related ripasudil/K-115 and the chemically divergent Y-27632 (Yamamoto et al., 2014 and reviewed in Abbhi and Piplani, 2018). However, selectivity is a challenge for these and other molecules. Structures of the kinase domain, which show high homology to other family members, in complex with fasudil, Y-27632 and other molecules have been reported (Li et al., 2012; Julian and Olson, 2014; Boland et al., 2015; Homan and Tesmer, 2015). Interestingly, a novel class of type I ROCK inhibitors was identified using fragment-based screening assisted by structure-guided design (Li et al., 2012). The ROCK2-preferring KD025/SLx-2119 or LYC-30937 may be interesting molecules given the predominant ROCK 2 expression in the CNS; and the hypothesis that ROCK1 may have a stronger relationship with vasodilation insensitivity and mortality (reviewed in Feng et al., 2016; Koch et al., 2018). BBB penetration optimization for ROCK inhibitors has not been disclosed though fasudil has been reported to alleviate EAE-dependent damage by decreasing BBB permeability (Huang et al., 2011).

#### Clinical Development

Fasudil and ripasudil are already in use or in clinical trials for pathological conditions including glaucoma, cerebral vasospasm, hypertension, atherosclerosis, and aortic stiffness (Julian and Olson, 2014). Despite beneficial effects, there are clinical limitations including poor oral bioavailability, blood pressure fluctuations and a relatively narrow safety window which makes them likely unsuitable for chronic dosing paradigms. Regardless, phase II clinical trials with fasudil have been initiated in ALS patients but results have not yet been reported (NCT01935518 and NCT03792490) (Koch et al., 2018; Lingor et al., 2019).

#### Synopsis

As for many other kinases, systemic inhibition of ROCK is expected to result in significant side effects. A molecule that avoids the blood pressure liabilities would be of great interest for clinical assessment in neurodegenerative disorders, however, to date this has proved very challenging.

## Summary

**FIGURE 3:**
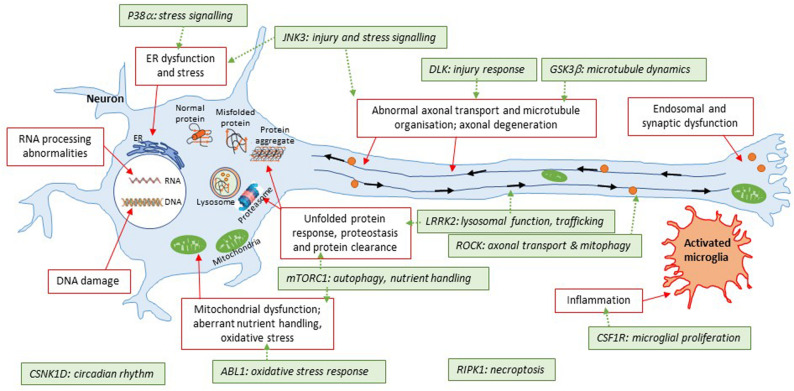
Illustration of kinase targets covered in this review with respect to converging pathways in neurodegeneration. This simplified schematic summarizes some of the common areas of pathophysiology in neurodegenerative diseases. The general theme is that the disease protein(s) are misfolded; which then triggers a number of downstream events such as protein aggregation, energy imbalances, altered proteostasis, stress responses to the unfolded proteins themselves or downstream consequences of events triggered by the unfolded proteins. Some of these pathways are further connected (e.g., diseased mitochondria can be removed by a specialized form of autophagy, known as mitophagy; furthermore, trafficking defects can apply to specific organelles illustrated here such as autolysosomes). The 11 kinases covered in this review have roles to play with respect to some of these pathways that have been shown to be altered. A key question for many targets is how closely related they are to initial triggers of pathophysiology; and whether inhibition of these targets will beneficially impact on multiple aspects of disease pathophysiology. LRRK2 is an exception here given that it is a *bona fide* genetic cause of Parkinson’s disease for a subset of patients.

The rationale behind protein kinases as relevant targets for CNS drug discovery is aligned with the appreciation that many cellular processes are tightly regulated by reversible phosphorylation processes; and that at least a number of these may become aberrant in disease (Figure 3). Interest in the potential of brain penetrant kinase inhibitors for neurodegenerative disease has grown in the last decade despite the dogma that brain penetrant, selective kinase inhibitors were not achievable.

The greatest challenge facing any CNS-targeted drug is effective penetration of the BBB and this is certainly not restricted to kinase inhibitors (Figure 2). At least some kinase inhibitors currently in the clinic appear to be opportunistic as opposed to the output of focused discovery and development workflows. Accordingly, the molecules in question have properties that do not align with those of CNS-penetrant compounds which raises concerns around the outcome of pending clinical trials with drugs that were developed and approved for indications requiring systemic exposure (reviewed in Shi and Mader, 2018). Indeed, a common concern when considering the current set of kinase inhibitors in clinical assessment is toxicity, which may be due at least in part to the suboptimal partitioning of the molecules in the human body, i.e., requirement of high systemic exposure to achieve therapeutically significant brain concentrations. With increased focused development of CNS-intended kinase inhibitors, we anticipate increased reporting of relevant pharmacokinetic properties commensurate with assessing appropriate compartmental exposure. This could include information such as unbound partition coefficient (K_*pu,u*_) data together with confirmation of target engagement with clinical relevance such as PET (Watterson et al., 2013; Prabhakaran et al., 2017; Wager et al., 2017). Indeed, intentionally designed kinase inhibitor candidates for CNS indications exemplified by the LRRK2 and DLK molecules have been reported in the context of detailed DMPK and safety assessments.

Another consistent theme in this review is the cross-reactivity seen with a range of kinase inhibitors although this is improving with kinase panel screening activities to assess selectivity (Uitdehaag et al., 2012). Caution still must be leveraged as the use of different ATP concentrations used during the functional assay, the format of the assay and the selection of representative kinases for assessment does not fully indicate the true extent of the selectivity profile.

Structurally guided approaches and/or fragment-based drug discovery are key approaches that can be leveraged which address many of these challenges. Kinases are eminently amenable to x-ray crystallography to generated detailed structural data that, together with biophysical and bioinformatic approaches, can be effectively leveraged to understand the three-dimensional binding mode of ligands and inform the rational optimization into potent and selective lead compounds with CNS drug-like properties (Chessari and Woodhead, 2009; Brooijmans et al., 2010; Mortenson et al., 2014). Structurally guided approaches are further augmented when performed in conjunction with unbiased fragment screening which has potential to identify novel chemotypes/scaffolds or allosteric sites. In addition, with the rapid development of cryoEM and related technologies we may be able to gain much greater insight into the role kinases play in regulating multiprotein complexes and subsequently how we may be able to drug these enzymes in a more precise manner.

## Author Contributions

CB and LD collected and reviewed the literature, wrote, and critically reviewed. Both authors contributed to the article and approved the submitted version.

## Conflict of Interest

CB is a paid employee of Dementia Discovery Fund Ltd., as the CSO of LoQus23 Therapeutics. LD is a paid employee of Cerevance Ltd.

## Footnotes

1 https://www.cureparkinsons.org.uk/mjff-press-release-nilo-pd

2 https://www.fiercebiotech.com/biotech/biogen-makes-another-alzheimer-s-bet-paying-pfizer-75m-for-early-phase-drug

3 https://www.denalitherapeutics.com/investors/press-release?id=7361

## Abbreviations

AD: Alzheimer’s disease
ALS: amyotrophic lateral sclerosis
ALSP: adult onset leukoencephalopathy with axonal spheroids and pigmented glia
ATP: adenosine triphosphate
BBB: blood brain barrier
CNS: central nervous system
DLB: dementia with Lewy bodies
DMPK: drug metabolism and pharmacokinetics
FTD: frontotemporal dementia
GTP: guanosine triphosphate
HD: Huntington’s disease
HTS: high throughput screen
Kp/K_*pu,u*_: Partition coefficient/unbound partition coefficient
MPTP/MPP+: 1-methyl-4-phenyl-1,2,3,6-tetrahydropyridine/1-methyl-4-phenylpyridinium
MS: multiple sclerosis
ND: neurodegenerative disease
NHP: non-human primate
NPC: Niemann-Pick disease type C
PD: Parkinson’s disease
PET: positron emission tomography
PK: pharmacokinetics
PSP: progressive supranuclear palsy
RTK: receptor tyrosine kinase.
